# Dapagliflozin Reduces Systemic Inflammation in Patients with Type 2 Diabetes Without Known Heart Failure

**DOI:** 10.21203/rs.3.rs-4132581/v1

**Published:** 2024-03-25

**Authors:** Dennis Wang, Anna Naumova, Daniel Isquith, Jaime Sapp, Kim Anh Huynh, Isabella Tucker, Niranjan Balu, Anna Voronyuk, Baocheng Chu, Karen Ordovas, Charles Maynard, Rong Tian, Xue-Qiao Zhao, Francis Kim

**Affiliations:** University of Washington; University of Washington; University of Washington; University of Washington; University of Washington; University of Washington; University of Washington; University of Washington; University of Washington; University of Washington; University of Washington; University of Washington; University of Washington; University of Washington

**Keywords:** Type 2 Diabetes, inflammation, IL-1B, PBMC respiration, CMRI, cardiac fibrosis, SGLT2 inhibitor

## Abstract

**Objective:**

Sodium glucose cotransporter 2 (SGLT2) inhibitors significantly improve cardiovascular outcomes in diabetic patients; however, the mechanism is unclear. We hypothesized that dapagliflozin improves cardiac outcomes via beneficial effects on systemic and cardiac inflammation and cardiac fibrosis.

**Research and Design Methods:**

This randomized placebo-controlled clinical trial enrolled 62 adult patients (mean age 62, 17% female) with type 2 diabetes (T2D) without known heart failure. Subjects were randomized to 12 months of daily 10 mg dapagliflozin or placebo. For all patients, blood/plasma samples and cardiac magnetic resonance imaging (CMRI) were obtained at time of randomization and at the end of 12 months. Systemic inflammation was assessed by plasma IL-1B, TNFα, IL-6 and ketone levels and PBMC mitochondrial respiration, an emerging marker of sterile inflammation. Cardiac fibrosis was assessed by T1 mapping to calculate extracellular volume fraction (ECV); cardiac tissue inflammation was assessed by T2 mapping.

**Results:**

Between the baseline and 12-month time point, plasma IL-1B was reduced (−1.8 pg/mL, P=0.003) while ketones were increased (0.26 mM, P=0.0001) in patients randomized to dapagliflozin. PBMC maximal oxygen consumption rate (OCR) decreased over the 12-month period in the placebo group but did not change in patients receiving dapagliflozin (−158.9 pmole/min/10^6^cells, P=0.0497 vs −45.2 pmole/min/10^6^cells, P=0.41), a finding consistent with an anti-inflammatory effect of SGLT2i. ECV and T2 relaxation time did not change in both study groups.

**Conclusion:**

This study demonstrates that 12 months of dapagliflozin reduces IL-1B mediated systemic inflammation but affect cardiac fibrosis in T2D.

**Clinical Trial.gov Registration:**

NCT03782259

## Introduction

Sodium glucose cotransporter 2 (SGLT2) inhibitors have unique characteristics to inhibit the reabsorption of glucose in kidneys, causing an increase in urinary glucose excretion and reduction of plasma glucose. In the EMPA-REG OUTCOME and CANVAS trials, SGLT2 inhibitors have been shown to significantly reduce cardiovascular events in T2D patients [1, 2]. These benefits are independent of glycemic control. While the mechanism by which SGLT2 inhibition improves cardiovascular outcomes in T2D remains elusive, there is ample evidence in pre-clinical and clinical studies to suggest that SGLT2 inhibition is associated with a reduction in inflammation [3, 4]. Recently, subgroup analysis of the CANTOS trial suggests that anti-interleukin (IL)-1B therapy may reduce heart failure (HF) hospitalizations in myocardial infarction (MI) patients with elevated high sensitivity C-reactive protein (CRP) [5], implicating IL-1B as a key mediator in cardiac inflammation. Experimental T2D animal models suggest that SGLT2 inhibitors increase the rate of glucose and fatty acid oxidation leading to an increase in circulating ketone levels, which was shown to inhibit NLRP3 inflammasome activation, resulting in reduced IL-1B production in macrophages [6]. Taken together, it is hypothesized that SGLT2 inhibitor’s effect in improving cardiac outcomes is mediated through antagonizing IL-1B. However, to date, there has been no double-blind placebo controlled randomized trials to demonstrate that SGLT2 inhibition reduces systemic IL-1B in T2D patients.

Cardiac fibrosis, which is known to adversely affect diastolic function [7, 8], plays an important role in the pathogenesis of diabetic cardiomyopathy [9]. Detailed pathological examinations of diabetic heart reveal myocardial hypertrophy, interstitial fibrosis, capillary endothelial changes, and capillary basal laminae thickening [10]. Cardiac MRI using T1-mapping is capable of quantifying myocardial extracellular volume (ECV), a surrogate of fibrosis, with excellent inter- and intra-observer variability and could, therefore, be potentially employed for investigations in diabetic cardiomyopathy [11]. A recent study [12] showed that ECV by T1-mapping increased as the duration of diabetes increased from 3 to 9 months in diabetic rabbits, consistent with the changes in myocardial fibrosis verified by pathology. In addition, a relatively new feature tracking technique for Cardiac Magnetic Resonance Imaging (CMRI) used for myocardial strain assessment can provide insights into the regional functional abnormalities that cannot be detected in global measurements of cardiac function. Further, T2-mapping can help to assess myocardial edema caused by injury and inflammation [13].

The aims of this double-blind placebo-controlled study were to investigate whether dapagliflozin treatment for 12 months could reduce systemic and myocardial inflammation and improve myocardial fibrosis in T2D patients.

## Research Design and Methods

### Study Setting and Patients

This double-blind, randomized trial assigned adults with T2D to placebo or 10 mg dapagliflozin daily for 12 months. The study began on 2/26/2019; initial screening of patients and follow up visits occurred at the Clinical Atherosclerosis Research Laboratory at Harborview Medical Center, University of Washington. The study was completed on 11/16/2022.

### Inclusion Criteria

Age > 18; T2D history > 5 years; Hemoglobin A1c (7–10%), glucose control medication: insulin, metformin, and/or sulfonylurea.

### Exclusion Criteria

Current use of SGLT2 inhibitor; hypersensitivity to SGLT2 inhibitor; contraindications to MRI; eGFR < 45 ml/min/1.73m^2^; unstable or progressing renal disease; SBP < 100 mmHg; severe hepatic disease (Child-Pugh Class C); active hepatitis B or C, CV disease within 3 months before enrollment (myocardial infarction; CABG, coronary intervention; NYHA Class IV heart failure; TIA; stroke, PAD); Bladder cancer; or high risk of DKA, high risk of fracture (osteoporosis, osteopenia).

### Enrollment and Randomization

**Figure 1:**
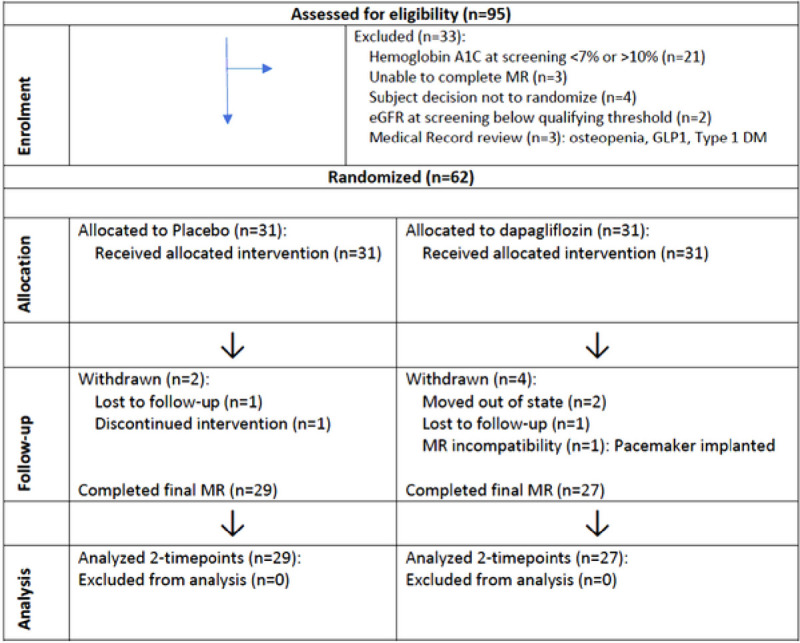
Trial CONSORT Flow Diagram MR, magnetic resonance; DM, diabetes mellites, GLP1, Glucagon-like peptide-1

T2D patients without known heart failure were enrolled in the study. During the enrollment period, 95 patients were assessed for eligibility, 62 patients were randomized, 56 patients completed the study protocol (Fig. 1). Randomization was stratified according to use of glucagon-like peptide (GLP-1) and angiotensin-II receptor blockers (ARBs).

### Study Intervention

Following screening visit and informed consent, patients were randomized 1:1 to placebo or 10 mg dapagliflozin daily for 12 months. Randomization was performed by the Investigational Drug Services at Harborview Medical Center. During the randomization visit, blood samples were collected for peripheral blood mononuclear cell (PBMC) respiration assessment and plasma samples were collected for cytokine and ketone measurements. Patients also received a baseline CMRI and laboratory evaluation. Patients had clinical visits at 3, 6, 9 months, and at the 12-month visit patients underwent a final CMRI along with blood and plasma sample collection.

### Outcomes

#### Primary outcomes:

Changes in global myocardial strain and ECV as assessed by T1 mapping (baseline to 12 months).

#### Secondary outcomes:

Changes in plasma IL-1B, TNFα, IL-6, IL-10, plasma ketones, T2 relaxation time (baseline to 12 months).

#### Exploratory outcome:

PBMC mitochondrial respiration

### Plasma Cytokine and Ketone Quantifications

Plasma samples were obtained from whole blood collected in EDTA-containing vacutainers post 2000g × 10’ at 4°C and stored in −80°C. Plasma concentrations of cytokines were determined by ELISA following manufacturer’s protocol (Biolegend): IL-1B (Cat: 437004), TNFα (Cat: 430204), IL-6 (Cat: 430504), and IL-10 (Cat: 430601). Plasma concentrations of β-hydroxybutyrate and acetoacetate were determined by EnzyChrom^™^ Ketone Body Assay Kit following manufacturer’s protocol (BioAssay Systems, Cat: EKBD-100).

### PBMC Oxygen Consumption Rate (OCR) Measurement

PBMC was isolated from whole blood collected in acid-citrate-dextrose vacutainers post density gradient (Histopaque-1077, Sigma-Aldrich Cat: 10771) centrifugation. Freshly isolated PBMCs were resuspended in Seahorse XF medium (Cat: 102353–100) and then plated (10^6^ cells per well) onto Seahorse XFe24 cell culture plate. PBMC mitochondrial respiratory function was assessed by measuring the oxygen consumption rate at basal and maximal stimulated conditions using Seahorse XFe24 Analyzer as described previously [14].

### Cardiac Magnetic Resonance Imaging

CMRI examination was done at a 3T clinical whole-body scanner (Ingenia, Phillips^®^) located at the BioMolecular Imaging Center (BMIC) at the University of Washington, South Lake Union campus. CMRI protocol included: steady state free precession (SSFP) cine imaging to measure heart LV chamber volumes (assessing dilatation and hypertrophy), contractile function and myocardial strain; naïve and post-contrast T1 mapping and ECV fraction to assess changes in diffused myocardial fibrosis; T2 mapping to assess myocardial inflammation; T2* mapping to assess iron deposition; Late gadolinium enhancement for visualize focal fibrosis.

All imaging acquisitions were done with ECG gating and breath hold technique. Imaging parameters are shown in **Supplemental Table 1**.

### Image Processing and Analysis

Volumetric LV analysis and analysis of quantitative maps (T1, T2, T2*) were performed using Philips IntelliSpace Portal (ISP) software. Volumetric parameters are reported as indexes, after adjustment for body surface area. Variables are compared to normal age specific ranges reported in the literature.

ECV maps were generated offline using MATLAB software. ECV was calculated from native and post-contrast T1 values for blood and myocardial tissue, the partition coefficient lambda (λ), and hematocrit using the following formulas: ECV = λ(1−hematocrit); λ = (1/T1 myocardium post-contrast-1/T1 myocardium-native)/(1/T1 blood post-contrast-1/T1 blood-native).

Feature tracking was performed using Circle Cardiovascular Software (cvi-42, Circle Cardiovascular Imaging Inc., Calgary, Alberta, Canada) to measure myocardial strain and strain rate from the bSSFP short-axis and long-axis cine images. Long-axis cine images were further used to compute global myocardial longitudinal strain. Short-axis images were used to compute circumferential and radial strain and strain rate. The global values were obtained through averaging the values according to an American Heart Association 17-segment model [15].

### Statistical Analysis

For systemic inflammatory endpoints (plasma cytokines, plasma ketones, and PBMC OCR), we compared baseline and 1-year post-intervention values in the dapagliflozin and placebo groups. P-values were determined by paired two-tailed t-test. Parametric t-test was used if distribution passes normality test, otherwise non-parametric t-test (Wilcoxon) was used.

For CMRI outcomes, we compared baseline characteristics in the dapagliflozin and placebo groups and expressed age as mean and standard deviation and categorical variables as numbers and percents. The difference between baseline and 1-year results was calculated for the primary outcomes in the dapagliflozin and placebo groups. Within each group, the difference between baseline and 1 year was assessed with the paired t-test. The differences between drug and placebo groups were compared with the Wilcoxon Rank Sum Test.

To adjust for multiple comparisons, the level of statistical significance was set at 0.0125 (.05/4) for primary outcomes, and 0.0083 (.05/6) for secondary outcomes.

### Ethical Oversight

The trial was approved by the Institutional Review Board at the University of Washington.

## Results

### Baseline Characteristics

As shown in Table 1, mean age of participants was 62 years and 17% were female. Of the participants, 61% had hypertension, 60% had hyperlipidemia, and 40% had a family history of coronary artery disease. No clinically meaningful differences were noted in the baseline characteristics between the dapagliflozin and placebo groups following randomization.

### Systemic Inflammatory Endpoints

**Figure 2:**
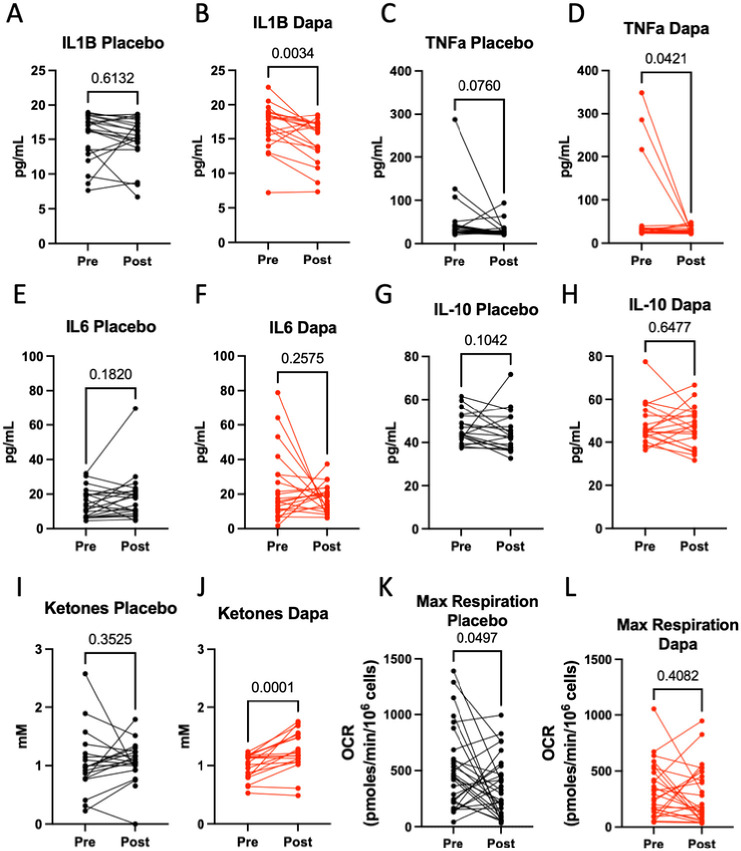
Dapagliflozin reduces systemic inflammation Levels of pre- and post-12-months treatment of placebo or dapagliflozin: A-H, Plasma IL-1B, TNFα, IL-6, and IL-10; I-J, Plasma ketones (acetoacetate + βhydroxybutyrate); K-L, PBMC maximal oxygen consumption rate (OCR). P-value determined by paired two-tailed T-test. Parametric t-test is used if distribution passes normality tests, otherwise non-parametric t-test is used. For plasma cytokines (IL-1B, TNFα, IL-6, and IL-10) and ketone, statistical significance set to P <= 0.0083 (0.05/6) with Bonferroni correction.

Between baseline and 1-year follow-up, circulating CRP did not change significantly in either dapagliflozin or the placebo group (Table 3). Among the plasma proinflammatory cytokines, we observed a significant reduction in IL-1B, but not TNFα or IL-6, in patients randomized to dapagliflozin (Figs. 2A–2F). IL-10, a known pro-fibrotic and anti-inflammatory cytokine, also did not change significantly in either group. (Figs. 2G and 2H).

Next, plasma ketones have been implicated as an important mediator for the beneficial effects associated with SGLT2 inhibitors, including anti-inflammation [16]. Here, we observed a significant increase of plasma ketones (β-hydroxybutyrate and acetoacetate) in patients randomized to dapagliflozin but not in the placebo group (Figs. 2I and 2J).

PBMC mitochondrial function is an emerging marker of sterile inflammation: a decline of PBMC maximal oxygen consumption rate (OCR), measured in the presence of mitochondrial uncoupler to facilitate maximal electron transport chain activity, is associated with increased pro-inflammatory cytokine expressions in chronic heart failure [14, 17]. Here, we observed that while the PBMC basal OCR did not change in either dapagliflozin or placebo group (**Supplemental Fig. 1**), the PBMC maximal OCR of patients receiving placebo declined while that of those receiving dapagliflozin did not change between baseline and 1-year follow-up (Figs. 2K and 2L), suggesting that SGLT2 inhibitor had an anti-inflammatory effect. Given PBMC is a major source of circulating cytokines, these results suggest that dapagliflozin may reduce systemic inflammation by acting on the PBMC – IL-1B axis, possibly mediated by ketones.

### Cardiac MR Endpoints

As shown in Table 2, ECV, a measurement of cardiac fibrosis, and myocardial strain values did not differ between baseline and at 1-year follow-up in either study group. The global radial peak strain in placebo group at 1-year trended higher in comparison to baseline (31.3 ± 10.4% vs. 27.3 ± 7.1%, P = 0.043, respectively). However, after adjusting for multiple comparisons (corrected standard P = 0.0125), the P-value of 0.043 was not statistically significant.

T2 relaxation time, a measurement of cardiac inflammation, of patients receiving placebo trended higher, suggesting worsening inflammation (48.6 ± 3.3 ms vs. 50.2 ± 3.8 ms, P = 0.045), between baseline and 1-year follow-up, while that of those receiving dapagliflozin did not change. However, the P-value of 0.045 was not statistically significant when compared to the corrected standard P = 0.0083 for multiple comparison. The representative CMRI of the same patient before intervention and at 1-year follow up are shown in **Supplemental Fig. 2**.

### Additional Endpoints

In comparison to the placebo group, patients randomized to dapagliflozin demonstrated significant reductions in random serum glucose (−33 mg/dl) vs (−3.48 mg/dl) (P = 0.006) and hemoglobin A1c (−0.52%) vs (0.11%) (P = 0.012) between baseline and 12-month follow-up. No differences were noted in brain natriuretic peptide (BNP) (Table 3)

## Conclusion

In this study, 12-months treatment of dapagliflozin reduced plasma IL-1B level. To our knowledge, this is the first time that SGLT2 inhibitor is shown to lower IL-1B in a placebo-controlled double-blind randomized clinical trial. However, dapagliflozin treatment did not result in a change in plasma IL6, plasma TNFα or serum CRP, consistent with the majority of prior clinical studies [18]. Furthermore, dapaglifozin significantly increased plasma ketones and attenuated the decline of PBMC maximal OCR, which was previously shown to inversely correlate with IL-1B expression in chronic heart failure [14, 17]. Despite the improvements in circulatory inflammatory endpoints, we did not observe significant changes in CMRI measurements of myocardial fibrosis and strain in patients receiving dapagliflozin. Of note, in the placebo group, there was a trend of worsening T2 relaxation time (inflammation), which was not observed in the dapagliflozin group, suggesting dapagliflozin may attenuate the progression of cardiac inflammation.

IL-1B is an inducible pro-inflammatory cytokine made primarily by immune cells, such as monocytes and macrophages, to function in cardiac repair as well as injury [19]. Under the NFkB-mediated transcriptional regulation, Pro-IL-1B is produced and stored intracellularly. Activation of NLRP3 inflammasome results in the cleavage and maturation of IL-1B prior to secretion. IL-1B has been shown to worsen myocardial contractile function and relaxation and induce hypertrophy [20–23]. Furthermore, in multiple animal studies, SGLT2 inhibitor is shown to antagonize the NLRP3-IL-1B axis to improve cardiovascular outcomes [4], substantiating IL-1B’s role as a potential mediator of cardiac inflammation. The recent CANTOS trial subgroup analysis provides clinical evidence that circulating IL-1B is not just a bystander but actively contributes to heart failure pathogenesis. The conjecture is corroborated by a number of small clinical trials using anakinra, a recombinant IL-1 receptor antagonist. In these studies, anakinra is shown to reduce CRP and HF hospitalization in acute STEMI patients [24, 25] and enhance exercise capacity and LVEF in HF patient with elevated CRP [26].

PBMC mitochondrial respiration is an emerging biomarker of sterile inflammation [14, 27–29], particularly in the setting of heart failure, and was previously shown to inversely correlate with the expressions of proinflammatory cytokines, such as IL-1B [14, 17]. It is postulated that upon stimulation by circulating mitochondrial damage associated molecular patterns (MitoDAMPs), PBMC produces IL-6, leading to mitochondrial dysfunction and mitochondrial ROS production in an autocrine manner [14]. Mitochondrial ROS subsequently activates the NLRP3 inflammasome, resulting in the maturation and release of IL-1B family of cytokines [30]. In the current study, we observed that SGLT2 inhibitor sustains mitochondrial oxidative capacity of circulating immune cells, supporting the notion that PBMC may be a source of circulating IL-1B, and a potential therapeutic target in T2D. Together, our results are consistent with the proposed mechanism that SGLT2 inhibitor lowers systemic inflammation by increasing plasma ketones, which acts on peripheral immune cells (PBMCs) to inhibit NLRP3 inflammasome activation and IL-1B production [6]. Recently, transcriptomic analyses indicate that disruption of mitochondrial pathways (TCA cycle and oxidative phosphorylation) in *circulating monocytes* is a marker of elevated cardiovascular risk in T2D patients [31]. Whether and how mitochondrial dysfunction in circulating monocytes plays a role in the pathogenesis of T2D and heart failure is an area of active research.

In addition to anti-inflammation, SGLT2 inhibitors have a wide range of effects on hemodynamic, neurohormonal, metabolic and endothelial function. There are several potential direct and indirect pathways leading to improvement of cardiac structure and function and myocardial substrate utilization in T2D. For example, SGLT2 inhibitors provide glycemic control, reduce blood pressure, reduce arterial stiffness, decrease body weight and reduce visceral adiposity, which could indirectly lead to improved cardiac function in T2D patients [32]. On the cellular level, SGLT2 inhibitors could directly affect cardiac function by reducing oxidative stress, attenuating myocardial fibrosis [33, 34]. In this study we did not find a significant effect of dapagliflozin on strain when compared to placebo. We also did not find a significant change in strain measures in the placebo control group over a 12-month period; raising the question whether the enrolled T2D patients truly had underlying structural heart disease.

Myocardial T1 mapping methods are used to measure tissue fibrosis and reflects a composite signal from intracellular and extracellular compartments; however, T1 measurements can be confounded by renal clearance, hematocrit, and type and dosage of gadolinium [35]. To adjust for these factors, the most widely accepted approach to determine fibrosis is to measure ECV which has been shown to be a good measure of interstitial fibrosis. In the current study, we found that 12 months of SGLT2 inhibition compared to placebo did not alter ECV of T2D patients. Our findings are consistent with a prospective study which enrolled 35 T2D subjects: Before and after CMRI was performed following 6 months of Empagliflozin showed no significant effect on ECV [36]. Furthermore, in our study, SGLT2 inhibition did not change the level of plasma IL-10, a pro-fibrotic cytokine. While these results do not support the notion that the cardioprotective effects of SGLT2 inhibition is mediated via improving cardiac fibrosis, further studies are required to unravel the mechanism of action by which SGLT2 inhibitors enhance cardiac outcomes.

There are several strengths of this study. First, there was excellent adherence with treatment as evidenced by significant reduction in blood glucose, hemoglobin A1c, and BMI in subjects randomized to dapagliflozin (Table 3). Second, the randomized study design was a strength, since many previous studies on SGLT2 inhibitors utilized a prospective study design. Third, the use of circulating inflammatory endpoints as well as CMRI allowed for the assessment of anti-inflammatory effect of SGLT2 inhibitor at both the systemic and organ levels.

There are several limitations and weaknesses. First, this is a relatively small clinical trial which was not powered to detect smaller effect sizes. For example, while there is a trend that dapagliflozin attenuates the progression of cardiac inflammation (T2 relaxation time), the study is underpowered to detect a statistically significant difference. Second, previous animal studies of SGLT2 inhibition utilized direct pathologic examination of inflammation and fibrosis, whereas CMRI are known to be less sensitive in assessing these parameters. Third, the 12-month treatment period may not have been sufficient to result in detectable cardiac structural and functional changes, although in a prospective study, 3 months of SGLT2 inhibition was sufficient to demonstrate improvement in diastolic function [37]. Fourth, we did not have a matched, non-diabetic control group. Fifth, higher ECV values (greater than 32%) are associated with worse outcomes in patients with known myocarditis [38]. In contrast, the T2D cohort in our study had baseline ECV of 27–28%. To address the possibility that patients with higher baseline ECV would derive more benefits from SGLT2 inhibition, we performed a subgroup analysis in patients in the upper half of ECV values at baseline (ECV ~ 30%); however, in this cohort (N = 17) we still did not find a significant difference in ECV between the drug and placebo groups.

In conclusion we demonstrated that 12 months of daily dapagliflozin in T2D patients reduces circulating IL-1B, increases plasma ketones, and prevents the decline of PBMC mitochondrial maximal respiration, but does not improve cardiac fibrosis or strain by CMRI.

## Figures and Tables

**Table 1:** Baseline characteristics

Characteristic	Dapagliflozin (n = 31)	Placebo (n = 31)
Age, (mean, standard deviation)	62 (9)	62 (11)
Women	6 (19%)	5 (16%)
Hispanic	1 (3%)	4 (13%)
**Race**		
White	23 (74%)	20 (64%)
Black	3 (10%)	3 (10%)
Asian	3 (10%)	6 (19%)
Native American	2 (6%)	1 (3%)
Not provided	0 (0%)	1 (3%)
Hypertension	19 (61%)	19 (61%)
Diabetes mellitus	31 (100%)	31 (100%)
Hyperlipidemia	21 (68%)	18 (58%)
Current smoker	2(6%)	3 (10%)
Family history MI	12 (39%)	11 (36%)
Family history stroke	14 (45%)	11 (36%)
History MI	1 (3%)	2 (6%)
Angina	1 (3%)	2 (6%)
Coronary artery bypass surgery	0 (0%)	2 (6%)
Percutaneous coronary intervention	3 (10%)	1 (3%)

*P* > 0.05 for all treatment group comparisons

MI, myocardial infarction.

**Table 2:** CMRI outcomes

	Drug			Placebo			Difference (1 year – baseline)		
Outcome	Baseline	1 year	P*	Baseline	1 year	P*	Drug	Placebo	P**
ECV	27.7 (2.9)	28.4 (2.0)	0.20	28.5 (2.1)	28.7 (2.3)	0.56	0.71 (2.75) (n = 26)	0.24 (2.16) (n = 28)	0.20
Radial peak strain global	31.2 (8.7)	33.9 (9.5)	0.14	27.3 (7.1)	31.3 (10.4)	0.043	2.71 (9.38) (n = 27)	4.01 (10.19) (n = 29)	0.80
Circumferential peak strain global	−17.8 (2.9)	−17.7 (3.6)	0.93	−15.7 (2.8)	−16.7 (2.9)	0.12	0.05 (3.21) (n = 27)	−0.97 (3.28) (n = 29)	0.43
Longitudinal peak strain global	−12.9 (3.4)	−12.0 (3.8)	0.27	−11.0 (3.9)	−11.3 (5.1)	0.81	0.88 (4.06) (n = 27)	−0.24 (5.32) (n = 29)	0.62
T2 relaxation time	50.5 (4.3)	50.0 (5.7)	0.70	48.6 (3.3)	50.2 (3.8)	0.045	−0.51 (6.59) (n = 25)	1.61 (4.14) (n = 29)	0.30

For ECV, radial, circumferential, and longitudinal peak strain measurements, statistical significance set to P < = 0.0125 with Bonferroni correction. For T2 relaxation time, statistical significance set to P < = 0.0083 with Bonferroni correction

**Table 3:** Additional Outcomes

	Drug			Placebo			Difference (1 year – baseline)		
Outcome	Baseline	1 year	P*	Baseline	1 year	P*	Drug	Placebo	P**
C-reactive protein	1.7 (1.6)	2.5 (4.1)	0.08	2.4 (0.5)	1.8 (0.3)	0.78	0.81 (2.95) (n = 28)	0.12 (2.20) (n = 29)	0.50
Glucose	159.8 (42.5)	126.8 (36.9)	0.004	164.5 (46.4)	161.0 (55.9)	0.64	−33.04 (55.08) (n = 28)	−3.48 (39.56) (n = 29)	0.006
A1c	7.9 (0.8)	7.4 (0.8)	0.007	7.8 (0.9)	8.0 (1.0)	0.63	−0.52 (0.95) (n = 28)	0.11 (1.17) (n = 28)	0.012
Hematocrit	42.5 (4.0)	40.9 (3.1)	0.08	41.4 (1.8)	41.4 (2.8)	0.96	−0.02 (0.05) (n = 26)	0.00 (0.02) (n = 29)	0.33
Body surface area	2.10 (0.21)	2.06 (0.21)	< 0.0001	2.05 (0.16)	2.04 (0.15)	0.14	−0.04 (0.04) (n = 26)	−0.01 (0.04) (n = 29)	0.005
BNP	37.1 (38.0)	55.1 (72.1)	0.08	31.9 (27.7)	36.4 (24.4)	0.31	17.96 (52.56) (n = 28)	4.58 (23.61) (n = 29)	0.94

All variables expressed as mean (standard deviation)

* by paired t-test.

** by Wilcoxon Rank Sum Test

BNP Brain natriuretic peptide.

## Data Availability

All data will be uploaded and made available to the public once the manuscript has been published.

## References

[1] Zinman B, Empagliflozin, Cardiovascular Outcomes, and Mortality in Type 2 Diabetes. N Engl J Med. 2015;373(22):2117–28. 26378978 10.1056/NEJMoa1504720

[2] Neal B, Canagliflozin and Cardiovascular and Renal Events in Type 2 Diabetes. N Engl J Med. 2017;377(7):644–57. 28605608 10.1056/NEJMoa1611925

[3] Lopaschuk GD, Verma S. Mechanisms of Cardiovascular Benefits of Sodium Glucose Co-Transporter 2 (SGLT2) Inhibitors: A State-of-the-Art Review. JACC Basic Transl Sci. 2020;5(6):632–44. 32613148 10.1016/j.jacbts.2020.02.004 PMC7315190

[4] Scisciola L, Anti-inflammatory role of SGLT2 inhibitors as part of their anti-atherosclerotic activity: Data from basic science and clinical trials. Front Cardiovasc Med. 2022;9:1008922. 36148061 10.3389/fcvm.2022.1008922 PMC9485634

[5] Ridker PM, Antiinflammatory Therapy with Canakinumab for Atherosclerotic Disease. N Engl J Med. 2017;377(12):1119–31. 28845751 10.1056/NEJMoa1707914

[6] Kim SR, SGLT2 inhibition modulates NLRP3 inflammasome activity via ketones and insulin in diabetes with cardiovascular disease. Nat Commun. 2020;11(1):2127. 32358544 10.1038/s41467-020-15983-6 PMC7195385

[7] de Souza RR. Aging of myocardial collagen. Biogerontology. 2002;3(6):325–35. 12510171 10.1023/a:1021312027486

[8] Zile MR, Myocardial stiffness in patients with heart failure and a preserved ejection fraction: contributions of collagen and titin. Circulation. 2015;131(14):1247–59. 25637629 10.1161/CIRCULATIONAHA.114.013215 PMC4390480

[9] Asbun J, Villarreal FJ. The pathogenesis of myocardial fibrosis in the setting of diabetic cardiomyopathy. J Am Coll Cardiol. 2006;47(4):693–700. 16487830 10.1016/j.jacc.2005.09.050

[10] Fischer VW, Barner HB, Larose LS. Pathomorphologic aspects of muscular tissue in diabetes mellitus. Hum Pathol. 1984;15(12):1127–36. 6238897 10.1016/s0046-8177(84)80307-x

[11] Taylor AJ, T1 Mapping: Basic Techniques and Clinical Applications. JACC Cardiovasc Imaging. 2016;9(1):67–81. 26762877 10.1016/j.jcmg.2015.11.005

[12] Zeng M, The Association between Diffuse Myocardial Fibrosis on Cardiac Magnetic Resonance T1 Mapping and Myocardial Dysfunction in Diabetic Rabbits. Sci Rep. 2017;7:44937. 28338005 10.1038/srep44937 PMC5364486

[13] Bohnen S Performance of t1 and t2 mapping cardiovascular magnetic resonance to detect active myocarditis in patients with recent-onset heart failure. Circ Cardiovasc Imaging, 2015. 8(6). 10.1161/CIRCIMAGING.114.003073 26015267

[14] Zhou B, Boosting NAD level suppresses inflammatory activation of PBMCs in heart failure. J Clin Invest. 2020;130(11):6054–63. 32790648 10.1172/JCI138538 PMC7598081

[15] Jung J, Patient-specific 17-segment myocardial modeling on a bull’s eye map. J Appl Clin Med Phys. 2016;17(5):453–65. 27685120 10.1120/jacmp.v17i5.6237 PMC5874123

[16] Lupsa BC, Kibbey RG, Inzucchi SE. Ketones: the double-edged sword of SGLT2 inhibitors? Diabetologia. 2023;66(1):23–32. 36255460 10.1007/s00125-022-05815-1

[17] Wang DD, Safety and Tolerability of Nicotinamide Riboside in Heart Failure With Reduced Ejection Fraction. JACC Basic Transl Sci. 2022;7(12):1183–96. 36644285 10.1016/j.jacbts.2022.06.012 PMC9831861

[18] Wang D, The effect of sodium-glucose cotransporter 2 inhibitors on biomarkers of inflammation: A systematic review and meta-analysis of randomized controlled trials. Front Pharmacol. 2022;13:1045235. 36467062 10.3389/fphar.2022.1045235 PMC9717685

[19] Bujak M, Frangogiannis NG. The role of IL-1 in the pathogenesis of heart disease. Arch Immunol Ther Exp (Warsz). 2009;57(3):165–76. 19479203 10.1007/s00005-009-0024-y PMC2788964

[20] Kumar A, Tumor necrosis factor alpha and interleukin 1beta are responsible for in vitro myocardial cell depression induced by human septic shock serum. J Exp Med. 1996;183(3):949–58. 8642298 10.1084/jem.183.3.949 PMC2192364

[21] Dai RP, Differential expression of cytokines in the rat heart in response to sustained volume overload. Eur J Heart Fail. 2004;6(6):693–703. 15542404 10.1016/j.ejheart.2003.11.014

[22] Busch K, Inhibition of the NLRP3/IL-1beta axis protects against sepsis-induced cardiomyopathy. J Cachexia Sarcopenia Muscle. 2021;12(6):1653–68. 34472725 10.1002/jcsm.12763 PMC8718055

[23] Peiro C, IL-1beta Inhibition in Cardiovascular Complications Associated to Diabetes Mellitus. Front Pharmacol. 2017;8:363. 28659798 10.3389/fphar.2017.00363 PMC5468794

[24] Abbate A Effects of interleukin-1 blockade with anakinra on adverse cardiac remodeling and heart failure after acute myocardial infarction [from the Virginia Commonwealth University-Anakinra Remodeling Trial (2) (VCU-ART2) pilot study]. Am J Cardiol, 2013. 111(10): p. 1394 – 400. 23453459 10.1016/j.amjcard.2013.01.287 PMC3644511

[25] Rommens PM, Goffin J. [Osteosynthesis of the dens axis fracture]. Acta Chir Belg, 1991. 91(4): p. 169 – 74. 1950299

[26] Abbate A, Interleukin-1 and the Inflammasome as Therapeutic Targets in Cardiovascular Disease. Circ Res. 2020;126(9):1260–80. 32324502 10.1161/CIRCRESAHA.120.315937 PMC8760628

[27] Shirakawa R, Mitochondrial reactive oxygen species generation in blood cells is associated with disease severity and exercise intolerance in heart failure patients. Sci Rep. 2019;9(1):14709. 31605012 10.1038/s41598-019-51298-3 PMC6789126

[28] Li P, Mitochondrial respiratory dysfunctions of blood mononuclear cells link with cardiac disturbance in patients with early-stage heart failure. Sci Rep. 2015;5:10229. 26018291 10.1038/srep10229 PMC4448851

[29] Sack MN. Mitochondrial fidelity and metabolic agility control immune cell fate and function. J Clin Invest. 2018;128(9):3651–61. 30059015 10.1172/JCI120845 PMC6118630

[30] Traba J, Fasting and refeeding differentially regulate NLRP3 inflammasome activation in human subjects. J Clin Invest. 2015;125(12):4592–600. 26529255 10.1172/JCI83260 PMC4665779

[31] Julla JB, Blood Monocyte Phenotype Is A Marker of Cardiovascular Risk in Type 2 Diabetes. Circ Res; 2023. 10.1161/CIRCRESAHA.123.322757 38152893

[32] Cowie MR, Fisher M. SGLT2 inhibitors: mechanisms of cardiovascular benefit beyond glycaemic control. Nat Rev Cardiol. 2020;17(12):761–72. 32665641 10.1038/s41569-020-0406-8

[33] Lin B, Glycemic control with empagliflozin, a novel selective SGLT2 inhibitor, ameliorates cardiovascular injury and cognitive dysfunction in obese and type 2 diabetic mice. Cardiovasc Diabetol. 2014;13:148. 25344694 10.1186/s12933-014-0148-1 PMC4219031

[34] Zhang N, Dapagliflozin improves left ventricular remodeling and aorta sympathetic tone in a pig model of heart failure with preserved ejection fraction. Cardiovasc Diabetol. 2019;18(1):107. 31429767 10.1186/s12933-019-0914-1 PMC6702744

[35] Puntmann VO, T1 Mapping in Characterizing Myocardial Disease: A Comprehensive Review. Circ Res. 2016;119(2):277–99. 27390332 10.1161/CIRCRESAHA.116.307974

[36] Hsu JC, Effect of Empagliflozin on Cardiac Function, Adiposity, and Diffuse Fibrosis in Patients with Type 2 Diabetes Mellitus. Sci Rep. 2019;9(1):15348. 31653956 10.1038/s41598-019-51949-5 PMC6814842

[37] Lan NSR, The effects of sodium-glucose cotransporter 2 inhibitors on left ventricular function: current evidence and future directions. ESC Heart Fail. 2019;6(5):927–35. 31400090 10.1002/ehf2.12505 PMC6816235

[38] Grani C, Prognostic Value of Cardiac Magnetic Resonance Tissue Characterization in Risk Stratifying Patients With Suspected Myocarditis. J Am Coll Cardiol. 2017;70(16):1964–76. 29025553 10.1016/j.jacc.2017.08.050 PMC6506846

